# Horner’s Syndrome: An Unusual Complication of Transcervical Zenker’s Diverticulectomy

**DOI:** 10.1155/crot/5656273

**Published:** 2026-08-17

**Authors:** Yue Ma, Caroline C. Jeffery, Edward J. Damrose

**Affiliations:** ^1^ Division of Laryngology, Department of Otolaryngology-Head and Neck Surgery, University of California, San Francisco, California, USA, berkeley.edu; ^2^ Faculty of Medicine and Dentistry, Surgery Department, University of Alberta, Edmonton, Alberta, Canada, ualberta.ca; ^3^ Division of Laryngology, Department of Otolaryngology-Head and Neck Surgery, Stanford University Medical Center, Stanford, California, USA, stanford.edu

**Keywords:** Horner’s syndrome, intraoperative complication, surgical injury, Zenker’s diverticulum

## Abstract

Horner’s syndrome is a clinical entity encountered by head and neck surgeons in patients with penetrating neck injury, post‐neck dissections, and cervical sympathetic chain schwannomas. However, Horner’s syndrome after transcervical resection of Zenker’s diverticulum has not been previously described in the published literature. A patient who had failed two previous attempts at endoscopic repair of a large Zenker’s diverticulum underwent transcervical diverticulectomy with cricopharyngeal myotomy. A previous history of anterior cervical fusion at C5 and C6 was also present. Postoperatively, he developed pupillary asymmetry, left upper eyelid ptosis, and ipsilateral anhydrosis. Ophthalmology consultation was initiated, and cocaine 10% solution drops applied to both eyes confirmed the diagnosis. His symptoms persisted at 2 years postsurgery. Horner’s syndrome is a potential complication of Zenker’s diverticulectomy due to the close proximity of the pouch base to the sympathetic chain. The likelihood of injury to the cervical sympathetic chain is also increased in the setting of previous neck surgery.

## 1. Introduction

Horner’s syndrome, also known as oculosympathetic paresis, results from interruption of the sympathetic nerve along its neuronal pathway originating from the hypothalamus and terminating in the eye. Presentation is characterized by ipsilateral pupilary miosis, palpebral ptosis, facial anhydrosis, and enophthalmos on the affected side. Horner’s syndrome may be seen in a variety of clinical situations encountered by otolaryngology, head and neck, surgeons. These include traumatic and nontraumatic carotid artery dissection [1, 2], resection of sympathetic chain schwannomas, neck dissections [3], and thyroidectomy [4–6]. Dissection at the middle cervical ganglion or immediately superior to it, as may been seen in thyroid or carotid artery surgery, is the most likely point of injury to the sympathetic chain.

Possible injury to the sympathetic chain during transcervical excision of pharyngeoesophageal diverticula has been postulated but never reported [7]. We describe a case of Horner’s syndrome after transcervical Zenker’s repair along with potential mechanisms for this complication.

## 2. Case Report

**FIGURE 1:**
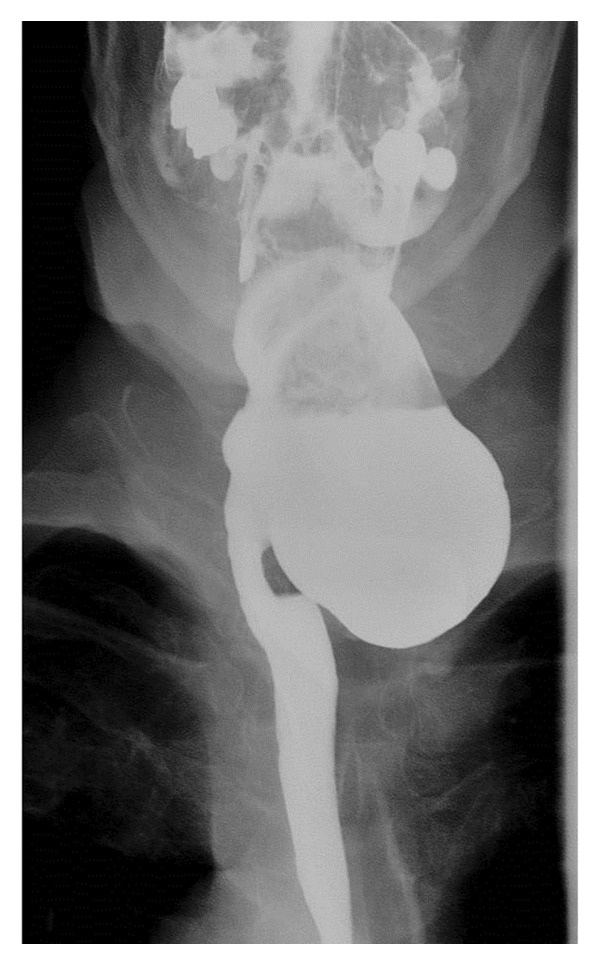
Barium esophagram demonstrating large recurrent Zenker’s diverticulum.

**FIGURE 2:**
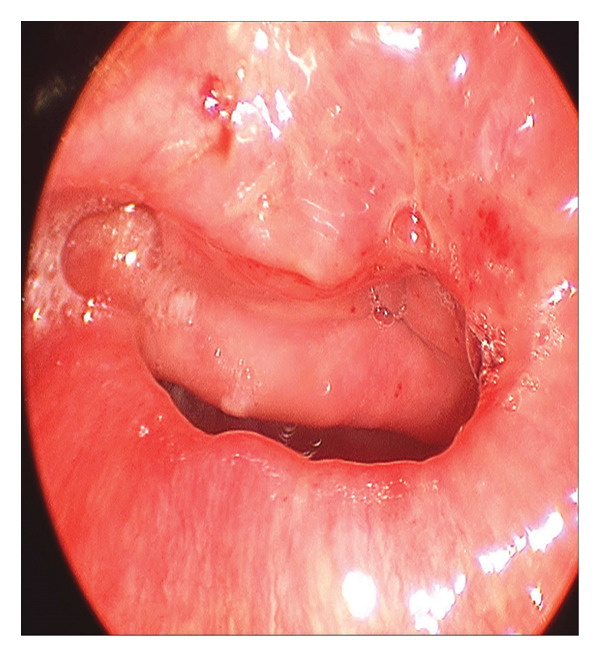
Intraoperative endoscopic view of diverticulum and cricopharyngeus muscle.

A 62‐year‐old male presented with dysphagia and a 5‐cm Zenker’s diverticulum. He had previously undergone two prior endoscopic diverticulotomies, the second of which was complicated by perforation. Barium swallow at the time of presentation to our service demonstrated a persistent Zenker’s diverticulum (Figures 1 and 2). The patient’s surgical history was significant for prior C5 and C6 anterior cervical discectomy and fusion via a left neck approach.

The patient underwent a standard transcervical diverticulectomy and cricopharyngeal myotomy via a left cervical approach. The sympathetic chain was neither encountered nor formally identified. The patient had scarring of the constrictor muscles to the prevertebral fascia secondary to prior surgery, but following dissection, the diverticulum was readily mobilized. Excision of the diverticulum and closure of the pharynx was performed with a reloadable 90‐mm linear stapler (PROXIMATE Reloadable Stapler TL90, Ethicon Endo‐Surgery, Cincinnati, OH, USA). There were no intraoperative complications, and blood loss was minimal.

On postoperative day one, the patient noticed asymmetry of his eyelids and pupils. He also complained of a burning sensation and blurred vision of the left eye. On examination, left eye ptosis and miosis were noted. His incision was intact, and his neck was otherwise unremarkable on inspection and palpation. Neurology and ophthalmology were consulted. In dim illumination, the patient’s right pupil diameter was 5.5 mm and the left was 3.0 mm. In bright illumination, the right pupil measured 3.5 mm and the left measured 2.5 mm. In addition to the clinical findings of left upper eyelid ptosis, anhydrosis, and pupillary miosis, pharmacologic testing using cocaine 10% solution was consistent with Horner’s syndrome, and a left Horner’s Syndrome was diagnosed. The patient’s blurred vision was felt to likely related to the miosis, while his burning sensation was felt likely related to decreased lacrimation, the later symptom being treated with artificial tears placed into the affected eye three times daily. At 2 years follow‐up, the patient had persistent symptoms and signs of Horner’s Syndrome.

## 3. Discussion

Horner’s syndrome results from interruption of the sympathetic nerve fibers anywhere along the oculosympathetic pathway originating from the hypothalamus and ending in the eye. The oculosympathetic pathway is composed of three ganglions and three neurons [8, 9]. The three ganglions are the cervicothoracic or stellate ganglion, the middle cervical ganglion, and the superior cervical ganglion. The stellate ganglion is the lowermost and is found anterior to the base of the transverse process of the C7. The middle cervical ganglion is the smallest and most variable in form, and its position is commonly located in close proximity to the inferior thyroid artery and the cricoid cartilage. The superior cervical ganglion is the largest and lies opposite the transverse process of C2 [9]. The central neuron originates from the hypothalamus and synapses at the level of C8‐T2 of the cervical spinal cord. The preganglionic neuron originates at the level of the C8‐T2 ventral roots and synapses in the superior cervical ganglion which is located near the angle of the mandible and the bifurcation of the common carotid artery [8]. The preganglionic neuron is involved in 84% of iatrogenic lesions [10]. The postganglionic neuron leaves the superior cervical ganglion to form a sympathetic plexus and travel with named blood branches of the internal carotid artery to innervate structures within the orbit, salivary glands, and cutaneous sweat glands. Within the orbit, the sympathetic branches innervate three major structures: dilator pupillae to dilate the pupil, Müller’s muscle to elevate the eyelid, and the lacrimal gland [8]. Thus, when the oculosympathetic pathway is interrupted, symptoms of ipsilateral pupillary mitosis, palpebral ptosis, and facial anhydrosis develop.

With the exception of cervical sympathectomy and surgical extirpation of tumors of the cervical sympathetic chain such as paragangliomas, schwannomas, and neurofibromas, Horner’s syndrome is a rare complication of head and neck surgeries [11]. In a retrospective study of 714 functional neck dissections, Horner’s syndrome was noted in only four cases [12]. In a review of 2208 thyroid and parathyroid procedures, Horner’s syndrome was seen in only six cases, for a reported incidence of 0.27% [13]. Thus far, no case of Horner’s syndrome after transcervical diverticulectomy has been published in the literature.

**FIGURE 3:**
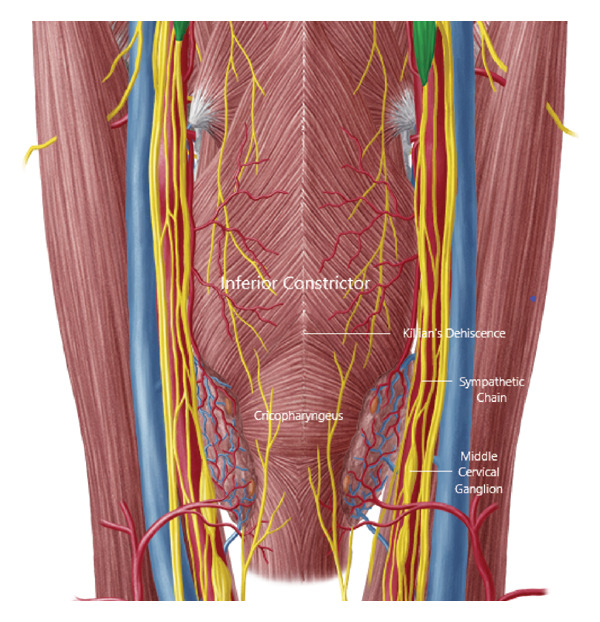
Anatomic relationships between the cervical sympathetic chain and Killian’s dehiscence.

From an anatomic point of view, Zenker’s diverticulum involves outpouching of mucosa through an area of dehiscence of weakness between the inferior constrictor muscle and the cricopharyngeus muscle (Figure 3). The pouch itself is usually situated just anterior to the longus colli muscle with the neck usually at C6. The middle or intermediate cervical ganglion is located posteromedial to the carotid sheath at C6 and seldom at C5 or C7 [14]. In addition, based on cadaveric studies, the middle cervical ganglion is on average 11.6 ± 1.6 mm from the medial border of the longus colli muscle [14]. Elbraheim et al. reported that the middle cervical ganglion is often just superior to the inferior thyroid artery [15]. Thus, the nerve is vulnerable when retracting the carotid sheath contents either medially or laterally, as is necessary in approaching Zenker’s pouch or in anterolateral approaches to the spine [14, 16]. This patient had a history of prior cervical spine fusion through an anterolateral approach, which may have increased the vulnerability of the nerve due to prior retraction on the carotid sheath and tissue fibrosis. Blood supply to the sympathetic chain is derived primarily from branches of the ascending pharyngeal artery, superior thyroid artery, and thyrocervical trunk [17]. This patient’s cervical sympathetic chain may have been more vulnerable to ischemia due to previous surgical dissection and disruption of vasculature. Formal identification of the sympathetic chain prior to significant retraction on the carotid sheath may aid in preventing such injuries, particularly in revision cases, while a deliberate more anterior approach favoring the wall of the diverticulum should be advocated as well. To clarify, while these aforementioned mechanisms of injury were considered in the context of the surgery and the patient’s history, the exact mechanism could not be confirmed.

While conventional intraoperative nerve monitoring is well established to mitigate the risk of injury to the vagus and recurrent laryngeal nerves, which are also at risk of injury in this location, no such monitoring currently exists to mitigate sympathetic chain injury. However, the use of laser doppler flowmetry has recently been described to evaluate changes in peripheral skin blood flow in patients undergoing lumbar sympathectomy [18]. Such technology, applied to the ipsilateral facial skin, could provide a means of monitoring increased blood flow, which could be an indication of impending nerve injury, as transmission to the blood vessels of the skin may be disrupted through traction or transection.

## Funding

No funding was received for this manuscript.

## Disclosure

Part of this work was presented as a poster at the American Bronchoesophagological Annual Meeting, May 12, 2013, Orlando, FL, USA.

## Consent

No written consent has been obtained from the patient as there is no patient identifiable data included. This research was reviewed and approved for publication by the Stanford University Human Subjects Research Institutional Review Board Protocol #88008.

## Conflicts of Interest

The authors declare no conflicts of interest.

## Data Availability

Data sharing is not applicable to this article as no datasets were generated or analyzed during the current study.
